# Tailored Carbon Nanocomposites for Efficient CO_2_ Capture

**DOI:** 10.3390/molecules30112408

**Published:** 2025-05-30

**Authors:** Diana Kichukova, Tsvetomila Lazarova, Genoveva Atanasova, Daniela Kovacheva, Ivanka Spassova

**Affiliations:** Institute of General and Inorganic Chemistry, Bulgarian Academy of Sciences, 1113 Sofia, Bulgaria; kichukova@svr.igic.bas.bg (D.K.); lazarova@svr.igic.bas.bg (T.L.); genoveva@svr.igic.bas.bg (G.A.); ispasova@svr.igic.bas.bg (I.S.)

**Keywords:** nanocarbon, reduced graphene oxide, CO_2_ adsorption, nanocomposite, carbon dots

## Abstract

CO_2_ capture by adsorption on proper solid materials appears to be a promising approach, due to its low energy requirements and ease of implementation. This study aimed to prepare efficient materials for CO_2_ capture based on composites of nanocarbon and reduced graphene oxide, using graphite, L-ascorbic acid, and glycine as precursors. The materials were characterized by XRD, low-temperature N_2_ adsorption, FTIR, Raman, and XPS spectroscopies, along with SEM and TEM. The CO_2_ adsorption capacities, heats of adsorption, and selectivity were determined. A hierarchical porous structure was found for NC-LAA, NC/RGO-LAA, and NC/RGO-Gly. At 273 K and 100 kPa, the adsorption capacities for NC-LAA and NC-Gly reached 2.6 mmol/g and 2.5 mmol/g, respectively, while for the composites, the capacities were 1.7 mmol/g for NC/RGO-Gly and 3.5 mmol/g for NC/RGO-LAA. The adsorption ability of the glycine-derived materials is related to the presence of nitrogen-containing functional groups. The heats of adsorption for NC-LAA, NC-Gly, and NC/RGO-Gly reveal chemisorption with CO_2_. Except for chemisorption, the NC/RGO-LAA material shows a sustained physical adsorption up to higher CO_2_ coverage. The best adsorption of CO_2_, observed for NC/RGO-LAA, is connected with the synergy between carbon dots and RGO. This composition ensures both sufficient oxygen surface functionalization and a proper hierarchical porous structure.

## 1. Introduction

Non-renewable fossil fuels remain the primary energy source, but their combustion releases significant amounts of carbon dioxide. Since the 1950s, this has been recognized as the leading human-induced factor contributing to atmospheric harm [1,2]. As a result, developing economically viable and efficient technologies for capturing, removing, or converting CO_2_ is increasingly crucial.

The main techniques proposed for the capture and utilization of CO_2_ are liquid absorption, adsorption, cryogenic fractionation, membrane separation, etc. Among them, the process of adsorption of CO_2_ on proper solid materials appears to be a promising approach, due to its low energy requirements and ease of implementation at low temperatures [3,4]. 

Various high-surface-area materials have been explored for CO_2_ capture, with recent studies focusing on different types of solid adsorbents [5,6,7,8,9,10,11,12]. These include mesoporous silica, natural and synthetic zeolites, MOFs [13,14], various carbon-based materials [15], etc. CO_2_ sorption in solid materials involves both physisorption and chemisorption, with the dominant process determined by the material’s characteristics and operating conditions (e.g., temperature, pressure, and gas composition). Physisorption relies on weak van der Waals forces and is favored at low temperatures and high pressures, while chemisorption involves stronger covalent or ionic bonds at specific surface sites. Most solids exhibit physisorption due to their high surface area and porosity, whereas chemisorption is often enhanced via functionalization to boost CO_2_ selectivity and uptake, especially at low partial pressures. The balance between these mechanisms governs overall sorption performance.

Some data on various sorbents, along with their respective CO_2_ adsorption capacities, are presented in Table 1 [12,15,16,17,18,19,20,21,22,23,24,25,26,27,28,29,30,31,32,33,34,35]. 

However, many of these materials suffer from drawbacks such as low adsorption capacity, poor selectivity, and slow saturation. Additionally, their practical use is often hindered by insufficient chemical, mechanical, or thermal stability. For some, their effectiveness decreases in humid conditions, further limiting their applicability [36,37].

Carbon nanomaterials (nanocarbons; NCs) are a class of carbon-based materials characterized by their nanoscale dimensions, typically ranging from a few nanometers to hundreds of nanometers in size. This category encompasses various forms of carbon, including carbon nanotubes, graphene, graphene oxide, fullerenes, carbon black, and activated carbon. These are promising adsorbents due to their low cost, eco-friendliness, and excellent physicochemical properties, including high surface area, tunable porosity, and stability [38,39,40,41]. They can be derived from various sources, including industrial and agricultural waste [42], and their surface functionality can be tailored via chemical modifications [42,43,44,45]. Recently, biopolymer-based carbon materials [46], lignin-based adsorbents [47], and solid sorbents like activated carbon, zeolites, metal–organic frameworks (MOFs), and porous organic polymers (POPs) have been evaluated for CO_2_ adsorption.

Traditional carbon materials like graphite and activated carbon have long been used, while newer forms such as fullerenes, carbon nanotubes, graphene, and carbon dots offer unique properties, enabling diverse applications [48,49]. Their versatility arises from carbon’s ability to form bonds in multiple structural configurations.

The 2D sp^2^-hybridized carbon framework comprises a group of new materials (carbon nanotubes, carbon nanorods, fullerenes, porous carbons, graphene, and reduced graphene oxide (RGO)). Their implementation in practice demands large-scale production, which is a technological challenge. This is why the development of new synthesis methods, especially top–down approaches, is the focus of researchers. These newly discovered carbon-based materials have been found to be applicable for the adsorption of many gaseous compounds, namely, for low-temperature adsorption, and especially for CO_2_ uptake [50]. Graphene-based materials have been successfully applied for CO_2_ capture. It has been found that the performance of such materials in CO_2_ sorption depends on the morphology and specific chemical functionalization ensuring high porosity and the presence of oxygen-containing surface groups [51,52,53,54]. 

Functional groups like hydroxyl, carboxyl, and epoxy on graphene oxide/reduced graphene oxide enhance their ability to interact with CO_2_ molecules, increasing their adsorption efficiency. Research suggests that CO_2_ adsorption on graphene-based materials occurs mainly through physisorption, which is influenced by their surface composition and structural defects in the graphene sheets [55]. Creating vacancies or defects in the graphene lattice can greatly enhance CO_2_ adsorption by generating localized states that strengthen interactions with gas molecules [56]. Computational studies have confirmed that defect sites on graphene surfaces enhance CO_2_ adsorption by increasing the adsorption energies, thereby improving the overall performance [56]. To improve the adsorption capacity of graphene-based materials, additional functionalization of the surface has been carried out with heteroatoms such as nitrogen and sulfur, functional groups, or the production of composites with polymers, silicas, or metal–organic frameworks [57,58,59,60,61].

Combining reduced graphene oxide with other types of carbon material can lead to synergistic effects that can significantly enhance the CO_2_ adsorption performance in several ways. The incorporation of other nanocarbons (carbon nanofibers, nanotubes, carbon dots, or metal-oxide-supported structures) could improve the textural properties and pore structure of RGO and provide functional groups that serve as active adsorption sites [62]. The combination of RGO with other nanocarbon structures, such as activated carbon nanofibers (ACNFs), has been shown to enhance the specific surface area and micropore volume of the adsorbents, which are directly correlated with enhanced CO_2_ adsorption capacity [63]. 

The production of carbon nanomaterials using green precursors presents notable advantages, including eco-friendliness and cost efficiency [64]. Moreover, it aligns with modern trends for sustainability by reducing dependence on hazardous chemicals and energy-intensive processes. The functionalization of nanocarbons by COOH- and NH_2_- groups allows for the enhancement of their CO_2_ affinity [65]. 

The modification of carbon materials with polar functional groups further boosts CO_2_ uptake from low-concentration streams, as reported in studies on acid-functionalized porous carbon [66,67]. Additionally, graphene nanocomposites offer tailored porosity and faster adsorption kinetics, making them viable for post-combustion CO_2_ capture [15,68]. Although promising, the application of carbon dots in CO_2_ capture is still in its early stages of investigation [69].

This study aimed to create efficient material for CO_2_ capture in a one-step synthesis of NC-RGO nanocomposites, using synthetic graphite and nitrogen (glycine) and oxygen-containing (L-ascorbic acid, L-AA) reducing agents as the initial materials. The latter two precursors were intended to simultaneously reduce the produced graphene oxide in situ and form additional nanocarbon material. The possible synergy between graphene’s surface area and tunable porosity and the functional adaptability of nanocarbons will be beneficial for optimizing these materials for environmental applications. The impact of the reducing agent type on the material’s surface chemistry and texture, related to the CO_2_ adsorption ability, will be investigated. 

## 2. Results and Discussion

The nanocarbons and composites under study are noted as bare reduced graphene oxide (RGO), nanocarbons prepared by L-AA (NC-L-AA) or glycine (NC-Gly), a composite prepared with the reducing agent L-AA (NC/RGO-LAA), and a composite prepared with the reducing agent glycine (NC/RGO-Gly).

### 2.1. XRD

**Figure 1:**
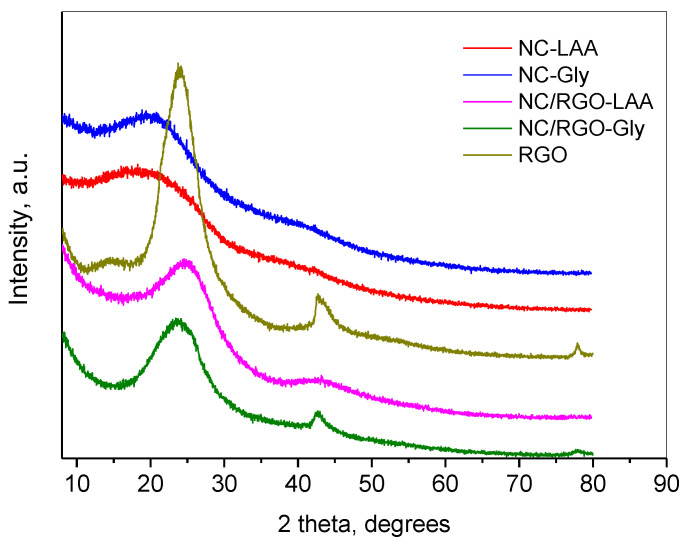
PXRD patterns of NC-LAA, NC-Gly, NC/RGO-LAA, NC/RGO-Gly, and referent RGO.

The XRD patterns of the initial graphite, LAA, and glycine are presented in Figure S1. A coincidence with the reference standard patterns PDF2-#00-008-1415, PDF2-#00-022-1560, and PDF2-#00-006-0230, respectively, was registered. The powder X-ray diffraction (PXRD) patterns of pure RGO, nanocarbon (NC) materials, and the respective composites (NC/RGO) can be seen in Figure 1. These patterns reflect the amorphous nature of the prepared NCs (blue and red) [70], exhibiting only very broad humps centered at around 20°2θ and 42°2θ, corresponding to interplanar distances of 4.43 Å and 2.15 Å, respectively. 

The pattern of RGO (dark yellow) contains a well-defined broad peak at 25.2^°^2θ (d = 3.52 Å), corresponding to (002) reflection of graphite, reflecting the slight widening of the spacing between graphene sheets. The (100) peak inside the graphene sheet is well visible at 42.7^°^2θ. 

The pattern of NC/RGO-LAA (magenta) shows more pronounced peaks at 25^°^2θ and 43.9^°^2θ (d = 3.55 Å and 2.06 Å), while the pattern of NC/RGO-Gly (dark green) consists of three well-defined peaks at 23.8^°^2θ, 42.7^°^2θ, and 77.7^°^2θ (d = 3.7 Å, 2.11 Å, and 1.23 Å). The well-pronounced peaks in the latter two patterns may be related to the presence of reduced graphene oxide, and these peaks can be attributed to the (002), (100), and (110) planes in the graphite-2H lattice. These two patterns combine the features of NC and RGO. 

### 2.2. Nitrogen Adsorption

**Figure 2:**
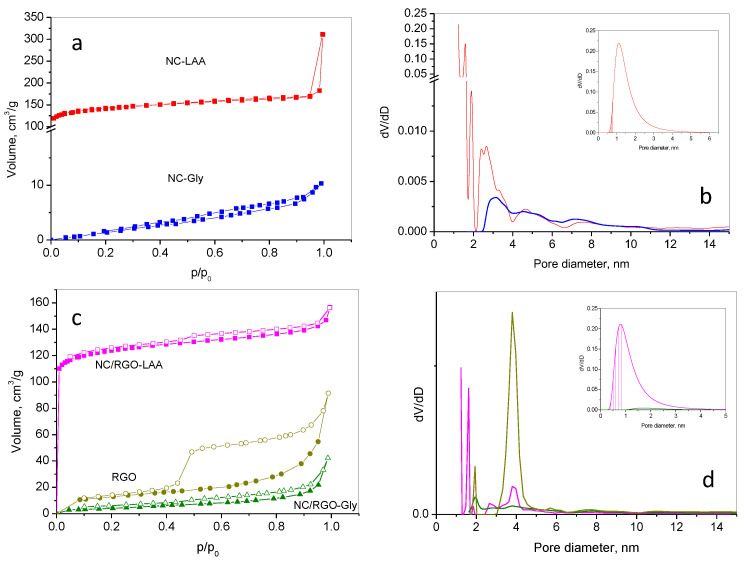
Nitrogen adsorption–desorption isotherms (**a**,**c**) and pore size distribution (**b**,**d**) of NC-LAA (red), NC-Gly (blue), NC/RGO-LAA (magenta), and NC/RGO-Gly (olive). The insets in (**b**,**d**) present the micropore size distribution of the respective samples. The shaded part of the insets represents the pores below 0.8 nm.

Nitrogen adsorption–desorption analysis was performed to study the surface and textural properties and to provide valuable information about the specific surface area, pore size distribution, and porosity of the materials prepared. The results are presented in Table 2 and Figure 2. 

The result for NC-LAA (red), according to the IUPAC classification [71], shows a mixed adsorption isotherm of types I (a steep initial rise in adsorption at low relative pressure and a plateau at higher pressures) and II (gradual uptake due to multilayer adsorption in mesopores/macropores), which usually occurs with a material of both microporous and mesoporous structures, leading to a combination of adsorption behaviors. There is no hysteresis. This suggests a hierarchical porous structure, confirmed by the high specific surface area and total pore volume, and by the significant presence of microporous volume (Table 1) in the sample. The pore size distribution supports this observation. NC-Gly (blue) shows an isotherm of type II (with no hysteresis), which indicates a material with non-porous or macroporous adsorption behavior. The specific surface area and total pore volume are not high, reflecting the particle aggregation and the absence of micropores. 

The composite material’s adsorption–desorption isotherms differ from those of the pure nanocarbon materials. Both NC/RGO-LAA (magenta) and NC/RGO-Gly (dark green) exhibit mixed type I and II isotherms, with a pronounced hysteresis (H3). This suggests that the composite materials have a non-rigid pore structure of aggregated plate-like particles with slit-like opened mesopores. A significant fraction of mesopores implies the presence of reduced graphene oxide. The specific surface area of NC/RGO-LAA is not as high as in NC-LAA, but the micropore volume is higher, with a larger content of micropores below 0.8 nm (inset). The content of pores below 0.8 nm was estimated to be 0.05 cm^3^ for NC/RGO-LAA and 0.005 cm^3^ for NC-LAA. Conversely, the specific surface area and pore volume of NC/RGO-Gly were higher than those of NC-Gly; additionally, a small amount of micropores appeared (Table 2, Figure 2, insets). 

The bare RGO sample (dark yellow) shows a type II–IV isotherm along with H3 type hysteresis. It possesses pronounced mesoporosity, with a negligible amount of micropores. Its specific surface area is not high, suggesting stacked graphene sheets or RGO agglomerates.

The texture analyses suggest that the pore structures of NC/RGO-LAA and NC/RGO-Gly combine the textural features of NC-LAA and NC-Gly with those of RGO, and new textural properties emerge.

### 2.3. SEM 

**Figure 3:**
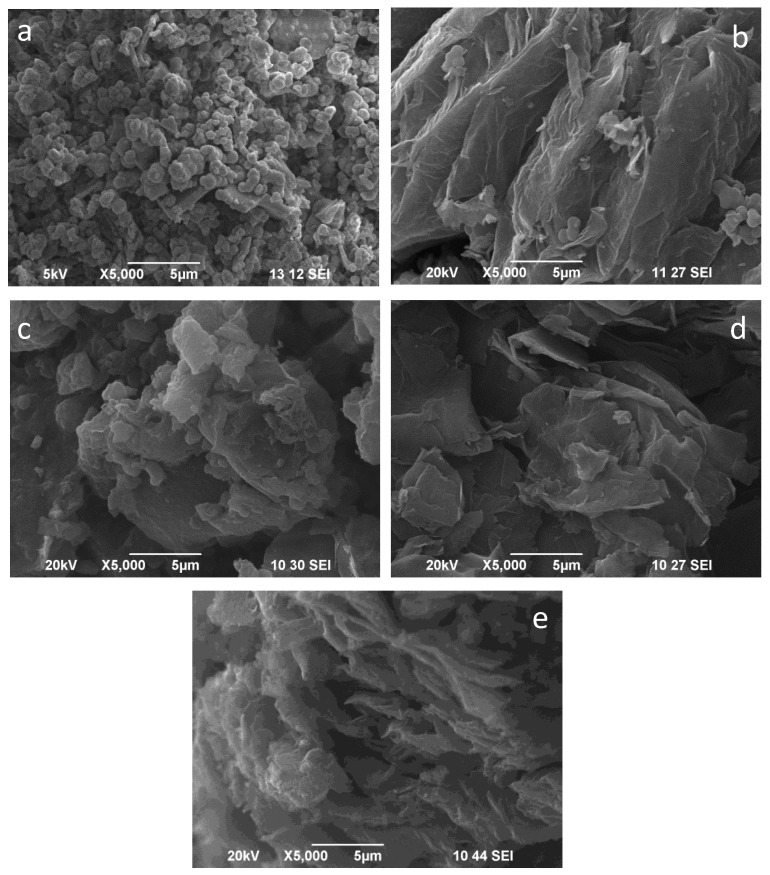
SEM (SEI) images of NC-LAA (**a**), NC/RGO-LAA (**b**), NC-Gly (**c**), NC/RGO-Gly (**d**) and RGO (**e**).

The Secondary Electron Imaging (SEI) pictures of the studied materials are presented in Figure 3. The referent RGO (Figure 3e) presents a typical, well-developed sheet morphology. The NCs obtained from two precursors show quite different morphologies. The NC-LAA material (Figure 3a) is built of round-shaped particles with similar sizes, loosely aggregated, and with a large number of voids between them. The approximate mean size (diameter) of the aggregates was evaluated to be less than 200 nm.

The observed morphology is a prerequisite for the developed porous structure registered by nitrogen physisorption studies. Conversely, the particles of the NC-Gly (Figure 3c) are irregularly shaped, exhibiting distinct edges and sizes ranging from nanometers to several microns, the latter being huge aggregates. As a result, the specific surface area is relatively small. The images of the NC/RGO-LAA show the features of both components of the composite (Figure 3b). One can see that the morphology of the reduced graphene oxide consists of several stacked graphene sheets, along with some ball-shaped particles, which represent the NC-LAA part of this composite, with particle aggregates of approx. 300 nm. The graphene sheets in the image of NC/RGO-Gly (Figure 3d) are more disrupted, forming smaller sheets, while the bigger sheets are wrinkled. The presence of NC-Gly in this sample is scarce and hardly distinguishable.

### 2.4. TEM

**Figure 4:**
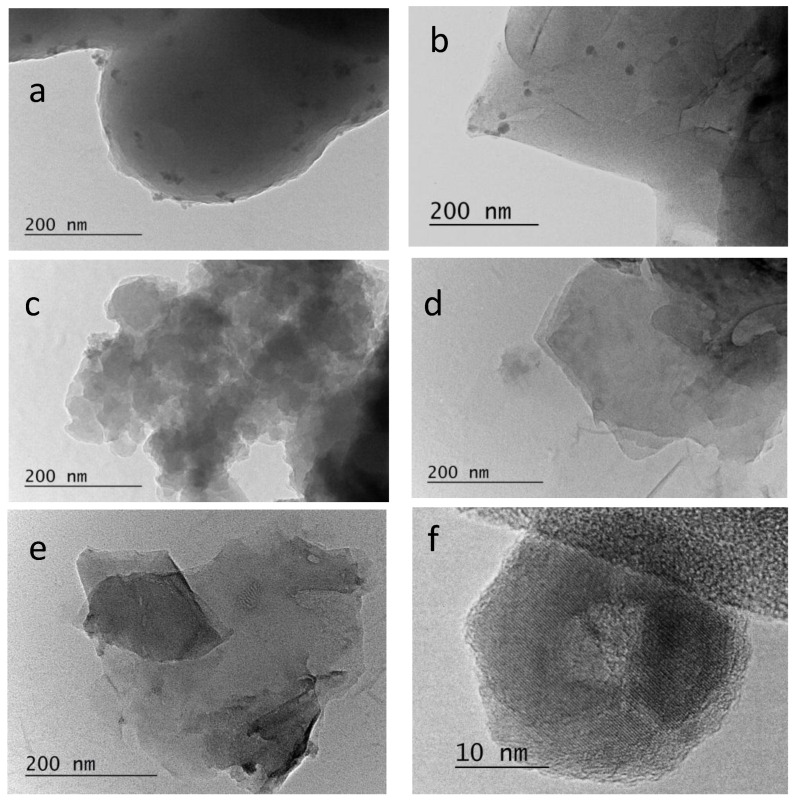
Bright-field TEM micrographs of NC-LAA (**a**), NC/RGO-LAA (**b**), NC-Gly (**c**), NC/RGO-Gly (**d**), and RGO (**e**), and HRTEM image of NC/RGO-LAA (**f**).

TEM images of all samples are presented in Figure 4. The referent RGO sample is presented as relatively small sheets, with some wrinkles and ragged edges. The NC-LAA contains amorphous mass covered with randomly distributed rounded small particles. 

The NC/RGO-LAA image presents a large graphene sheet, which is also covered with well-defined, small, spherical, but clustered particles. This observation correlates well with the findings in the SEM analyses. It was determined that the average size of the particles of both LAA-derived samples was about 11.5 nm. The inset in Figure 4b represents an HRTEM of one of these particles, showing its core–shell structure. This observation leads us to the assumption that these particles represent carbon dots or dot-like objects. Carbon dots are an interesting class of non-traditional carbon materials composed of sp^2^ and sp^3^ domains, with a high capacity to retain various oxygen-containing groups on their surface, making a core–shell structure. These nanocarbon materials show specific morphological characteristics (spherical shape, and size below 20 nm or even 10 nm, with a carbonic core and oxygen-functionalized shell), enabling surface tuning [72]. The combination of the specific morphological forms of carbon dots and RGO could prevent restacking of the graphene sheets, thus leading to enhanced specific surface area of the composite, and providing large exposure of functional groups of both types of carbon materials. 

On the other hand, the glycine-derived NC-Gly sample exhibits an amorphous material with irregularly shaped particles of no distinct sizes, as also seen in the SEM images. The image of NC/RGO-Gly shows predominantly large graphene sheets. Obviously, in this case, the NC material does not interact strongly with the RGO, and only a small part of the nanocarbon particles remains on the surface of the RGO sheets. This observation was further confirmed by the XPS analysis of the nitrogen content, estimating the nanocarbon content in the composite to be about 10%. 

### 2.5. FTIR and Raman

FTIR and Raman analyses were conducted to examine the structural peculiarities of the materials obtained and the possible interaction of the two components of the composites. The main peaks observed provide insights into their chemical structures and the changes that occur during the preparation process. The comparison of the spectra of the nanocarbons and the composites revealed changes in the intensity of certain absorption peaks.

**Figure 5:**
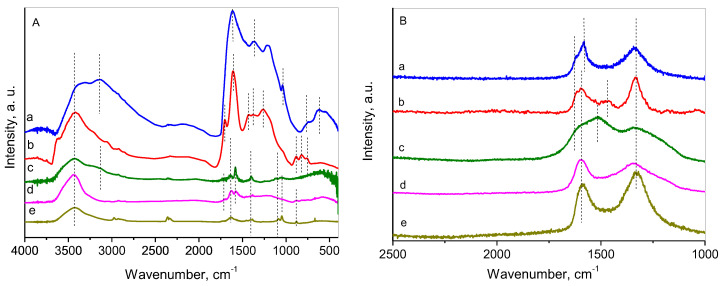
FTIR (**A**) and Raman spectra (**B**) of (a) NC-Gly (blue), (b) NC-LAA (red), (c) NC/RGO-Gly (olive), (d) NC/RGO-LAA (magenta), and (e) bare RGO (dark yellow).

The FTIR and Raman spectra of the nanocarbon materials and their composites with RGO are presented in Figure 5. Additionally, information on the FTIR of L-AA and glycine is shown in Figure S2 and Figure S3, respectively, corresponding to the spectra given in the literature [73,74]. The first glance at the FTIR spectra (Figure 5A) reveals the distinct difference between the nanocarbons and the composites, demonstrated mainly in the peak intensities within the whole range. The spectrum of NC-Gly (blue) contains two intensive but broad peaks at 3400 and 3144 cm⁻^1^, corresponding to O–H and N–H stretching vibrations, respectively, indicating the presence of hydroxyl (-OH) or amine (-NH) groups, possibly from functionalized carbon or residual moisture [75]. The triplet at the region 2964–2852 cm⁻^1^ is assigned to C–H stretching vibrations, suggesting aliphatic carbon (sp^3^-hybridized) from amorphous carbon or functionalized carbon structures [76]. This region of the spectrum of NC-LAA (red) represents a narrower peak of O–H stretching vibrations and does not show a line for N–H. The triplet, assigned to the C–H stretching vibrations of sp^3^-hybridized carbon, is also observed. Similar to the NC-Gly, the peaks of O–H and N–H stretching vibrations, as well as the triplet of C–H stretching vibrations, suggesting aliphatic carbon, are observed in the spectrum of NC/RGO-Gly (dark green), with lower intensities. A similarity is also registered between the spectra of NC-LAA and NC/RGO-LAA (magenta). 

A substantial difference is observed between the spectra of NC-Gly and NC-LAA in the range 1700–1000 cm^−1^. For NC-Gly, the peaks observed are at 1616 cm⁻^1^ for C=C stretching, characteristic of sp^2^-hybridized carbon; at 1360 cm⁻^1^ for C–O stretching from carboxyl or hydroxyl groups or C–N stretching in aromatic systems; at 1212 cm⁻^1^ for C–O stretching in oxygen-containing functional groups such as epoxide, carboxyl, or hydroxyl functionalities or C–N stretching in aliphatic amines; and at 1037 cm⁻^1^—C–O–C stretching vibrations, often linked to ether or epoxy groups [77]. For NC-LAA the spectrum presents peaks at 1702 cm^−1^, assigned to C=O stretching in carboxyl, ketone, and quinone groups; at 1612 cm^−1^, attributed to C=C stretching; at 1427 cm^−1^, due to C=C skeletal vibrations or C–O stretching; and at 1262 cm^−1^, linked to C–O stretching of epoxy or ether functionalities. Differences are also observed in the fingerprint region. For NC-LAA, the peaks in the range 890–760 cm^−1^ are attributed to C–H out-of-plane bending vibrations, while the peaks in the region 770–620 cm^−1^ for NC-Gly are assigned to C–N out-of-plane and C–N–C bending vibrations. 

In the FTIR spectra of RGO (dark yellow), a broad band centered at approximately 3430 cm⁻^1^ is attributed to the O–H stretching vibrations of water molecules. The diminished intensity observed here implies a partial removal of surface-adsorbed water during the thermal treatment. The peak observed at 1630 cm⁻^1^, corresponding to C=C stretching vibrations, together with the doublet between 2800 and 3000 cm⁻^1^, suggests the retention of the conjugated carbon basal planes in RGO. Additionally, the bands at 1370 cm⁻^1^, 1080 cm⁻^1^, and 1050 cm⁻^1^ can be attributed to C−OH bending, alkoxy C−O stretching, and C−OH stretching vibrations, respectively. It is visible that the intensities of the peaks of the composites (magenta and dark green) in the upper region are quite lower than those of the NCs. In this region, the peaks related to the RGO component of the composites are predominant, while those related to the NC are suppressed. For both spectra, the line at 1730 cm^−1^ indicates a strong carbonyl (-C=O) presence, possibly from carboxyl or ester groups, characteristic of oxidized carbon materials [78]. A second common peak at 1580 cm^−1^ is further confirmation of sp^2^-hybridized carbon in graphitic regions of RGO [79]. 

Raman spectroscopy provides structural information about the sp^2^ and sp^3^ domains in the carbon materials (Figure 5b). All of the materials show two peaks in the 1750–1000 cm^−1^ region. These correspond to the G band, associated with graphitic C=C stretching vibrations, and the D band, arising from defects, edge effects, and sp^3^ carbon structures. For the referent RGO sample (dark yellow), the G band is observed at 1596 cm⁻^1^, and the D band appears at 1338 cm⁻^1^, typical for RGO [80]. Comparison of the Raman spectra of NC-Gly (blue) and NC-LAA (red) reveals that both nanocarbons exhibit a complex G band, which includes a D′ component associated with in-plane defect-activated vibrations of the graphene sheets. The D band is broader for NC-Gly, implying smaller particles or an amorphous state. The intermediate peak at 1470 cm^−1^ for NC-LAA suggests surface functionalization or disorder beyond the basic D/G modes and is often linked to doping or surface peculiarities. The Raman bands at ~1100 cm⁻^1^ and ~1400 cm⁻^1^ are not characteristic of highly ordered carbon materials like graphene or carbon nanotubes. However, they could be observed in defective, functionalized, or partially disordered carbon materials, especially those with polyene-type (conjugated sp^2^ carbon) segments. They could originate from finite-length, disordered, polyene-type sp^2^ carbon structures, and possibly from C–C/C–O modes associated with functionalized or partially oxidized graphene-like materials. Their presence supports the idea of fragmented or chemically modified nanocarbons. 

As concerns the composite materials NC/RGO-Gly (dark green) and NC/RGO-LAA (magenta), the D and G bands are very broad and may be regarded as a superposition of several contributions. The linewidth of these Raman peaks is directly related to the degree of disorder, and broader linewidths typically indicate greater disorder in the carbon structure. The increase in linewidth and intensity in the D band, which is associated with defects and disorder in sp^2^ carbon systems, usually reflects more structural defects or amorphization. The G band, related to the in-plane vibration of sp^2^ carbon atoms, also broadens and shifts with increasing disorder. For the G band, the main contribution is from the presence of ordered sp^2^ carbon domains, and an additional D’ band at 1631 cm^−1^ is connected to the defect-induced mode or stress in the carbon lattice, distinct from the main D band. The observation of a D’ band at 1631 cm^−1^ as a shoulder of the G band is a characteristic feature that reflects the disordered nature of the material—specifically, the presence of defects and small sp^2^ clusters within the structure of the amorphous carbon material [81]. Both spectra show a very broad Raman D band, but the origin of the broadening seems different. In the case of NC/RGO-Gly, it is due to the nitrogen doping or amorphous carbon components, while for NC/RGO-LAA the broadening is possibly due to small graphitic domains, which are common in carbon dots due to their confined structure.

### 2.6. XPS

XPS analysis was used for elucidation of the surface chemical states. In the wide scan at 0–1100 eV, the photoelectron peaks of carbon C1s, oxygen O1s, and nitrogen N1s, the Auger peak O KLL of oxygen, and the corresponding X-ray satellite peaks were identified. The C1s region offers insights into the various chemical environments of carbon atoms in a material. Accurate deconvolution of the C1s envelope is critical for distinguishing between the bonding configurations. 

**Figure 6:**
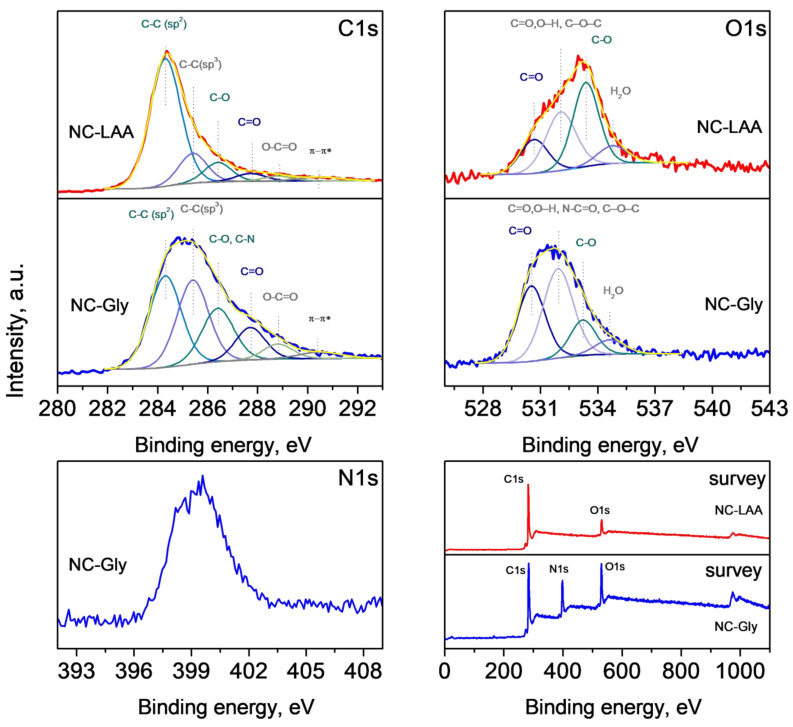
XPS C1s, O1s, and survey spectra of NC-LAA, NC/RGO-LAA, NC-Gly, and NC/RGO-Gly, and N1s spectra of NC-Gly and NC/RGO-Gly.

**Figure 7:**
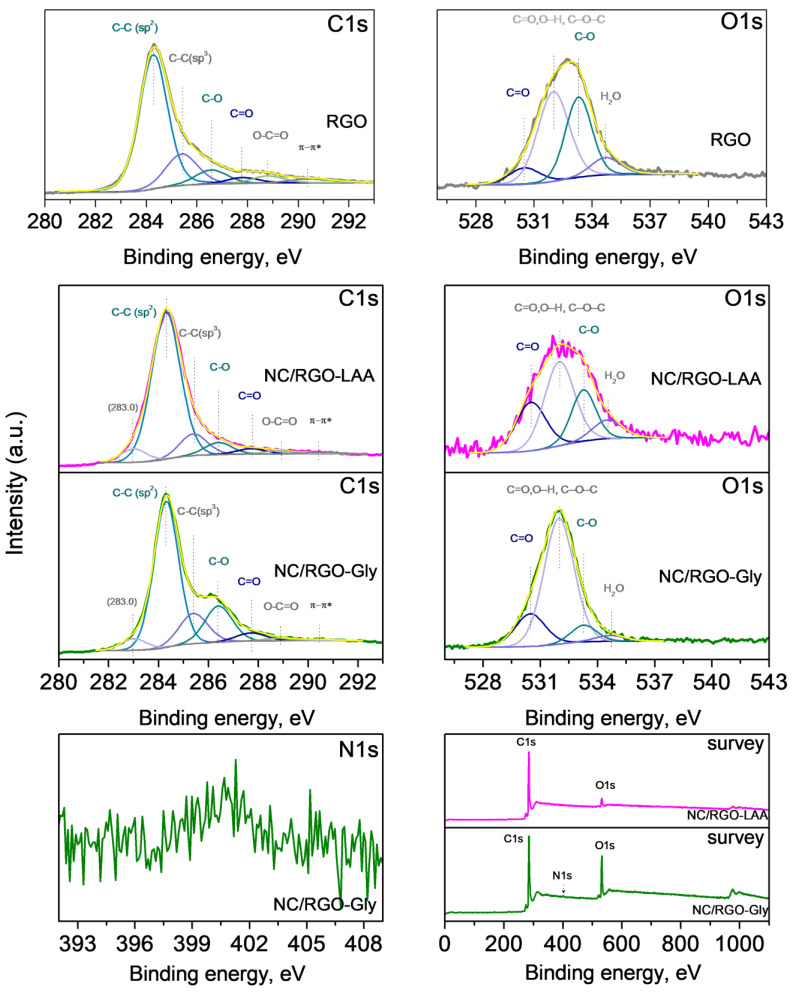
XPS C1s, O1s, and survey spectra of NC/RGO-LAA, NC/RGO-Gly, and RGO, and N1s of NC/RGO-Gly.

Figure 6 and Figure 7 present the C1s, O1s, and survey spectra for NC-LAA, NC-Gly, NC/RGO-LAA, NC/RGO-Gly, and the reference RGO, along with the N1s spectrum of NC-Gly and NC/RGO-Gly. Table 2 presents the surface chemical composition of the studied materials. 

The C1s region of all materials shows broad asymmetric peaks, due to the contribution of various carbon bonds. The careful deconvolution of the peaks reveals the main feature at 284.3 eV, belonging to sp^2^-hybridized carbon, characteristic of graphitic carbon, aromatic systems, etc. This peak was used for the calibration of the spectra, as the adventitious carbon is a contribution that cannot be distinguished. The contribution at 285.4 eV is often assigned to sp^3^-hybridized carbon atoms in aliphatic C–C and C–H bonds. One could notice that NC-Gly presents a higher peak for sp^3^-hybridized carbon, which is expected due to the aliphatic nature of the precursor glycine, while this peak is much lower for NC-LAA. The peaks at 286.4 eV and 287.7 eV could be ascribed to C–O (in alcohols and ethers) and C=O (in ketones or carboxylic derivatives), respectively. The peak at 286.4 eV also reflects the presence of C–N bonds in NC-Gly [82]. The peak at 288.8 eV is usually associated with O–C=O groups, and that at 290.5 eV represents a π–π* shake-up satellite that appears in highly conjugated π systems [83,84].

The O1s spectra of both nanocarbons confirm the presence of oxidized species such as C=O (530.5 eV), O–H, C–O–C (532.0 eV), C–O (533.3 eV), and H_2_O (534.6 eV). In the case of NC-Gly, a contribution of N-C=O at 532.0 eV could be suggested [82].

The spectra presented in Figure 7 for the composites and RGO show the same features at the same binding energies, differing only in their intensities. The small contribution in the C1s spectra of NC/RGO-LAA and NC/RGO-Gly at 283.0 eV likely originates from the presence of very small graphene particles, which exhibit different electrostatic charging during photoemission [85,86]. The N1s spectra definitively confirm the presence of nitrogen atoms in NC-Gly and NC/RGO-Gly (as detected by FTIR), although their content differs significantly in the samples, being 22.3 at.% and 2.1 at.%, respectively. Thus, it can be assumed that the content of nanocarbon in NC/RGO-Gly is approximately 10%. For the NC/RGO-LAA sample, the nanocarbon content could not be assessed, as both components contain the same chemical elements. The N1s peak at 399.3 eV in NC-Gly’s spectrum is well defined but is asymmetric, which indicates nitrogen atoms involved in different types of functional groups (pyrrolic, amine) [87]. The assignment of the N1s XPS peak at approximately 400.6 eV in NC/RGO-Gly is to graphitic (or quaternary) nitrogen, resulting from the substitution of a carbon atom by a nitrogen atom within the graphitic basal plane of the reduced graphene oxide [88].

The data presented in Table 3 reveal the contribution of each component of the composites in comparison to bare RGO. It is evident that the oxygen content is enhanced in the NC/RGO-LAA and NC/RGO-Gly due to the presence of nanocarbons. In the NC/RGO-Gly, the O/N ratio is also enhanced. 

### 2.7. CO_2_ Adsorption 

**Figure 8:**
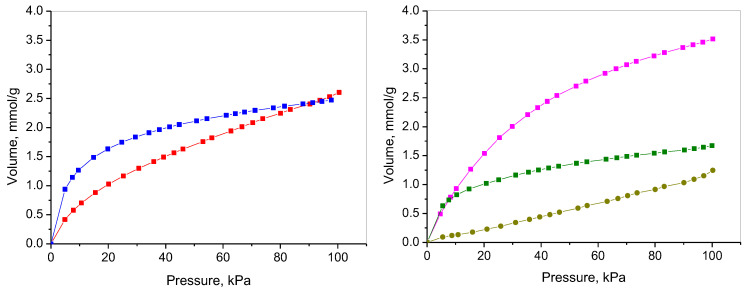
Adsorption isotherms of CO_2_ at 273 K for NC-LAA (left, red), NC-Gly (left, blue), NC/RGO-LAA (right, magenta), NC/RGO-Gly (right, olive), and bare RGO (right, dark yellow).

Figure 8 presents the adsorption of CO_2_ on the prepared NCs and NC/RGO composites. At 273 K and 100 kPa, the observed adsorption capacities for NC-LAA and NC-Gly reached 2.6 mmol/g and 2.5 mmol/g, respectively. The registered adsorption capacities for the composites were 3.5 mmol/g and 1.7 mmol/g for NC/RGO-LAA and NC/RGO-Gly, respectively. The referent bare RGO, prepared by the same procedure without L-AA and glycine, presented an adsorption capacity of 1.2 mmol/g. 

Recent studies have drawn attention to the relationship between CO_2_ uptake and total surface area, emphasizing the complexity of CO_2_ adsorption mechanisms beyond conventional pore size classifications. While the total BET surface area is commonly regarded as an important factor affecting gas adsorption capacity, emerging research suggests that this correlation is more nuanced than traditionally assumed. Factors other than surface area, such as the material’s porosity and the chemical properties of the adsorbent, play critical roles in the adsorption process, highlighting that a high BET surface area does not always translate into effective CO_2_ capture [89].

The CO_2_ adsorption is influenced not only by the total surface area but also by how that surface area is distributed among various pore sizes. A higher BET surface area is generally favorable, but the chemical environment and the specific porous architecture may more profoundly influence the CO_2_ uptake efficiency. In our case, NC-LAA possessed the highest BET surface area (496 m^2^/g), while NC-Gly had the lowest (15 m^2^/g), but their adsorption capacities were not proportional to them. In general, the adsorption capacities of the carbon-based materials are not due to their high specific surface area but, rather, a result of the volume of narrow micropores. Micropores significantly influence the adsorption behavior of CO_2_ due to their structural characteristics, and the volume and size distribution of these micropores are crucial for the CO_2_ capture efficiency of various carbon materials. Studies have emphasized the importance of narrow micropores in CO_2_ adsorption, as their dimensions closely match the kinetic diameter of CO_2_ molecules, thus strengthening van der Waals interactions, which, in turn, boosts the adsorption capacity. Consequently, materials possessing a higher volume of such micropores typically exhibit greater CO_2_ uptake [90,91]. It was found that the pore volume of micropores smaller than 0.8 nm (ultramicropores) is particularly influential in CO_2_ adsorption, especially under varying pressures [92,93,94], while larger micropores are filled with increased pressure. The ability of CO_2_ molecules to fill these small micropores is attributed to their strong adsorption potentials, which are enhanced in narrow micropores. In addition to volume, the specific surface area of microporous carbon materials also contributes to their CO_2_ adsorption capabilities. Furthermore, the interaction between CO_2_ molecules and the carbon surface, influenced by the micropore structure, can enhance adsorption through mechanisms such as hydrogen bonding and π–π interactions [95].

The different courses of the adsorption curves for NC-LAA and NC-Gly should be noted. At low pressures, NC-Gly adsorbs quite a lot of carbon dioxide, irrespective of its low micropore volume. As glycine is a nitrogen-containing compound, the prepared NC contains some amount of nitrogen in the form of basic functional groups, which can be registered from its XPS and FTIR spectra. In this case, glycine-derived materials may be regarded as N-doped carbon materials, leading to their enhanced CO_2_ affinity. The final adsorption capacities of both NC materials are almost equal, at 100kPa. 

It was reported that graphene sheets exhibited higher adsorption capacities compared to other carbon nanostructures, such as activated carbons and carbon nanotubes, under similar conditions, underlining the importance of the textural properties and surface functionalities in the adsorption performance of graphene derivatives [55]. However, our reference RGO sample exhibited the lowest adsorption capacity compared to the NC and NC/RGO materials. Analyzing the CO_2_ adsorption curves of the bare RGO, nanocarbons, and composites, we can deduce that composites show enhanced adsorption capacity values surpassing the simple sum of their components (in the case of NC/RGO-Gly, the nanocarbon content is 10%); thus, a synergic effect between the nanocarbons and RGO can be considered. For NC/RGO composite materials, a significant difference in the CO_2_ adsorption properties is registered. This fact is connected mainly to the differences in their textural characteristics. The NC/RGO-LAA composite material forms an interconnected network that facilitates gas diffusion (due to the presence of mesoporosity), combining the advantages of both reduced graphene oxide and nanocarbons (comprised of amorphous carbon and carbon dots), thus providing abundant active sites for the efficient physisorption of CO_2_ molecules [96]. It contains a large number of micropores (30% of them ultramicropores, below 0.8 nm) that play a role as active sites for CO_2_ adsorption. Hence, the combination of both types of carbon material ensures the specific surface functionalization and morphological features, enabling better CO_2_ adsorption. On the other hand, the NC/RGO-Gly shows a low micropore volume, which explains its lower capacity. It is worth mentioning that NC-Gly retained more nitrogen functionalities than NC/RGO-Gly, and this could explain its higher CO_2_ adsorption even with a lower specific surface area and microporosity. 

**Figure 9:**
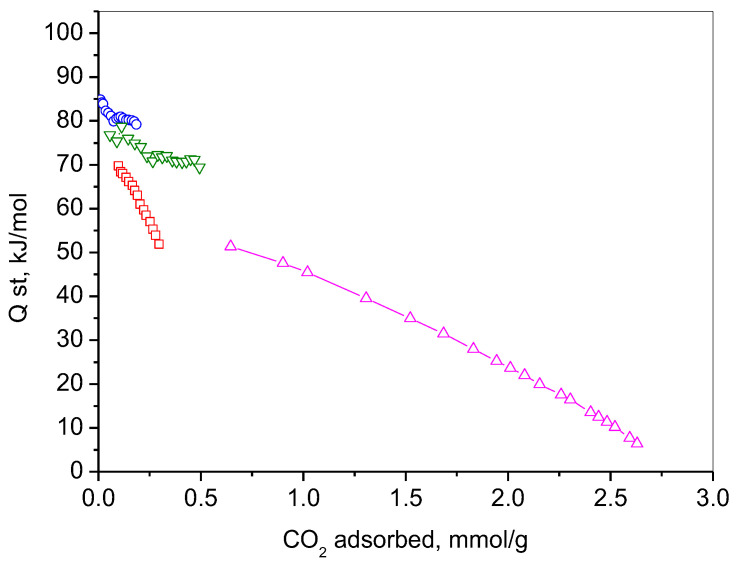
Heats of CO_2_ adsorption for NC-LAA (red), NC-Gly (blue), NC/RGO-LAA (magenta), and NC/RGO-Gly (olive).

The adsorption behavior of the studied materials could be explained further by presenting their isosteric heats of adsorption (Q_st_), as on carbon-containing materials (like activated carbon, biochar, carbon nanotubes, graphene oxide, etc.) they vary depending on the material’s surface chemistry, porosity, and structure. This indicates the strength of the interaction between CO_2_ molecules and the surface of the adsorbent. The calculated isosteric heats, according to the Clausius–Clapeyron equation from adsorption isotherms at different temperatures, are presented in Figure 9.

The heat of adsorption for physical adsorption is generally low, within the range of 8–40 kJ/mol [97]. This is consistent with values comparable to the heat of condensation of the adsorbate, reflecting weak van der Waals interactions. Conversely, chemisorption exhibits significantly higher heats of adsorption, ranging from 40 to 800 kJ/mol, indicative of stronger chemical bonding, and aligning with the enthalpy changes associated with chemical reactions [98]. It can be seen that all investigated samples at low CO_2_ coverage present high adsorption heats in the range of chemisorption. The results confirm the assumption that NC-Gly and NC/RGO-Gly adsorb CO_2_ through their nitrogen-containing groups. NC-LAA and NC/RGO-LAA have lower heats of adsorption compared to the glycine-derived materials, but they are still in a chemisorption range due to their oxygen surface functionalities. 

The NC/RGO-LAA, showing the highest adsorption capacity, combines initial chemisorption followed by physisorption. The composite NC/RGO-LAA benefits from a composite structure that combines hierarchical porosity with functional groups arising from incomplete reduction (see FTIR and XPS), promoting selective CO_2_ binding. The probable presence of carbon dots relies on the optimized formation of ultramicropores during preparation processes, creating an ideal environment for the physisorption of CO_2_. Some studies highlight that the design of carbon-based CO_2_ adsorbents must consider both the microstructural properties arising from the material’s precursor and the specific preparation methods used to tune their porosity and surface modification [55,99].

**Figure 10:**
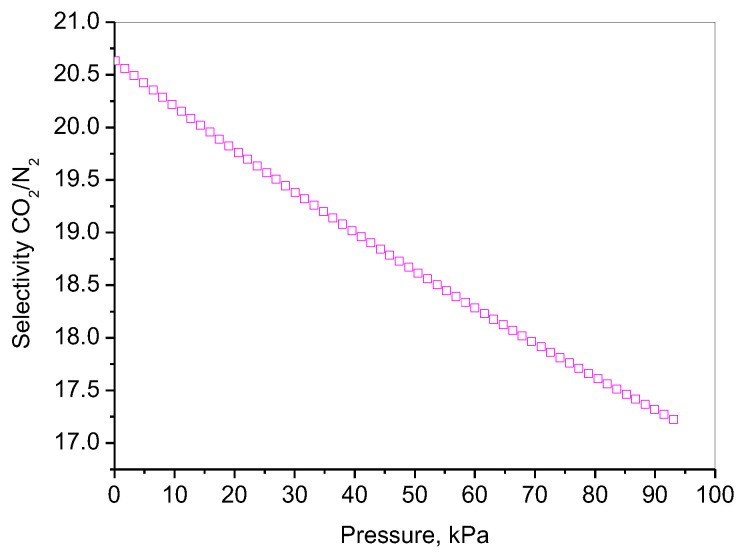
Predictive CO_2_/N_2_ selectivity of NC/RGO-LAA at 273 K, depending on pressure.

The effectiveness of carbon capture using solid adsorbents largely depends on their ability to selectively separate CO_2_ from N_2_. Because of this, post-combustion CO_2_ capture technologies require sorbents with high CO_2_/N_2_ selectivity. Typically, flue gas consists of 15% CO_2_ and a minor presence of water vapor, with N_2_ being the main component in it. The selectivity of an adsorbent material for CO_2_ over N_2_ is primarily motivated by differences in their physical and chemical properties and how they interact with the adsorbent’s surface. The predictive selectivity towards CO_2_ and N_2_ for the best-performing material (NC/RGO-LAA) was evaluated by separately measuring the adsorption capacity of pure CO_2_ and pure N_2_ at 0 °C using the IAST [100] and presented in Figure 10. 

The selectivity of an adsorbent for CO_2_ over N_2_ is driven by differences in molecular properties, including size, polarizability, electrostatic interactions, and adsorption energy [101,102,103]. At low pressures, CO_2_, which has a higher quadrupole moment (oxygen-containing) [104] and greater polarizability than N_2_ [105,106], preferentially adsorbs onto the NC/RGO-LAA due to interactions with functional groups (e.g., hydroxyl, carboxyl, etc.). The selectivity (CO_2_/N_2_) is usually high because CO_2_ interacts more strongly with the surface, and N_2_ adsorption remains weak. At intermediate pressures, competition with N_2_ may begin. As the NC/RGO-LAA material has high microporosity, the selectivity can remain relatively high. At higher pressures, the selectivity decreases as N_2_ adsorption increases, due to physical adsorption in larger pores. The NC/RGO-LAA material seems to show an optimized pore structure, with appropriate basic functional groups facilitating the electrostatic interactions with CO_2_, thus enhancing the selectivity over N_2_.

## 3. Materials and Methods

### 3.1. Materials

The materials used were synthetic graphite powder (Sigma-Aldrich, Saint Louis, MO, USA, 99% carbon, 50 mesh), H_2_SO_4_ (98%), KMnO_4_, H_3_PO_4_ (85%), and p.a. from Merck KGaA, Darmstadt, Germany. 

### 3.2. Synthesis of Composites

The synthesis of the composites started with the use of the modified Tour procedure for obtaining GO [107]. According to this, to 1 g of graphite powder previously ground in an agate mortar, 36 mL of H_2_SO_4_ and 4 mL of H_3_PO_4_ were added. The components were stirred intensively with a mechanical stirrer in an ice bath for 15 min. Then, 5 g of KMnO_4_ in 100 mL of distilled H_2_O was added dropwise to the mixture to avoid an increase in the temperature of the reaction mixture. This combination of strong acid and oxidizing agent ensures the effective oxidation of the graphite compound, providing exfoliation of the graphite sheets and pillaring them with oxygen-containing functional groups. Stirring was continued for another 30 min in the ice bath. After that, 50 mL of deionized water was added. The resulting suspension was left to stand for 12 h. As a next step, a solution of 5 mL of 30% hydrogen peroxide in 70 mL of deionized water was slowly added under constant stirring at room temperature to stop the oxidation. Then, 50 g of two types of reducing agent—one of them pure low-molecular-weight carbohydrate L-ascorbic acid (C_6_H_8_O_6_, L-AA), and the second one the simplest stable nitrogen-containing amino acid glycine (C_2_H_5_NO_2_, Gly)—was added directly to the suspension. In this synthesis step, part of the reducing agent provides in situ reduction of the GO to RGO, and the remaining content interacts with the aforementioned strong acids and KMnO_4_, resulting in dehydration and carbonization to a form of nanocarbon. During this stage, a certain amount of gases (CO_2_, SO_2_, O_2_, H_2_O) is released, although the gas evolution is not intensive. In the case of glycine, additional gases like NH_3_ or NO_2_ could be emitted. Then, the mixtures were sonicated for 15 min using a Sonix ultrasonic processor (20 KHz, 750 W) and left to stand for 2 h. A washing procedure was applied on a Buchner funnel until the drained water reaches pH = 6.5. The wet solid residue was dried at 363 K for 3 h. The resulting powder was subjected to additional thermal treatment at 873 K in Ar flow for 3 h. This step was intended to achieve full carbonization, accompanied by an increase in the specific surface area of the material.

The preparation scheme is presented in Figure S4.

### 3.3. Synthesis of Nanocarbons

The synthesis of NC-LAA and NC-Gly was performed in the following way: To the initial mixture of the abovementioned quantities of H_2_SO_4_ and H_3_PO_4,_ stirred for 15 min, a solution of 5 g of KMnO_4_ in 100 mL of distilled water was poured. Then, 50 g of L-ascorbic acid (L-AA) or 50 g of glycine was added. In this case (in the absence of graphite), the chemical reactions included only the interaction between the strong oxidants and the organic components. The mixture was stirred for 30 min in an ice bath, followed by the addition of 50 mL of deionized water. After standing for 12 h, a 5 mL hydrogen peroxide solution was slowly added. The sonication of the mixture was performed for 15 min, and after being left for 2 h it was washed to pH = 6.5 and dried at 363 K for 3 h. The additional thermal treatment was carried out at 873 K for 3 h in Ar flow. 

### 3.4. Synthesis of RGO

For comparison, bare RGO was prepared as described in Section 3.2, without using reducing agents. The obtained GO was washed to pH = 6.5 and dried at 363 K for 3 h. The reduction of GO was performed thermally at 873 K for 3 h in Ar flow.

### 3.5. Characterization

X-ray diffraction (XRD) analyses were carried out to determine the phase composition of the prepared materials. А Bruker D8 Advance powder X-ray diffractometer (Bruker AXS, Karlsruhe, Germany) with Ni-filtered Cu Kα radiation and a LynxEye solid-state position-sensitive detector was applied. The PDF-2 (2021) database of the International Center for Data Diffraction (ICDD) and the DiffracPlus EVA software package (version 4.1, Bruker AXS, Karlsruhe, Germany) were used to perform the analyses. The samples were placed on a zero-background sample holder from the standard set of the diffractometer accessories. 

The morphology of the prepared materials was examined using scanning electron microscopy (SEM) on a JEOL JSM-6390 (JEOL Ltd., Tokyo, Japan) with EDS (Oxford Instruments, Abingdon, UK). The samples were placed on a double-sided conductive copper tape. The particle diameter size evaluation was carried out on the basis of about 200 particles (aggregates) for each sample, using ImageJ (v. 1.51) (NIH, Bethesda, MD, USA).

The transmission electron microscopy (TEM) was carried out on a JEOL JEM 2100 microscope (JEOL Ltd., Tokyo, Japan) at 200 kV. High-resolution (HRTEM) mode was also used for observations of the structure of the samples. The samples were gently ground in an agate mortar and ultrasonically dispersed in ethanol for 15 min. A drop of the suspension was deposited onto a holey carbon film on a copper grid.

The texture properties of the synthesized materials were analyzed using the nitrogen adsorption isotherms obtained at −196 °C, performed on a Quantachrome Nova 1200e instrument (Anton Paar, Boynton Beach, FL, USA). The specific surface area was calculated using the BET method, while the total pore volume was determined at a p/p_0_ ≈ 0.99. The average pore diameter and pore size distributions were calculated using the NLDFT method with the slit pore model (equilibrium kernel). The volumes of the micropores were determined by the V-t method, and the micropore distributions were evaluated by the Dubinin–Astakhov method. 

A micro-Raman spectrometer (LabRAM HR800, HORIBA Jobin Yvon IBH Ltd. Glasgow, UK), configured with a 600 mm⁻^1^ diffraction grating and integrated optical microscopy, was employed for spectral acquisition. Excitation was provided by a 633 nm helium–neon (He–Ne) laser operating at an output power of 40 μW. A 100× objective lens was used to achieve precise laser focusing onto the sample surface. The samples were placed on a glass holder.

The Fourier-transform infrared (FTIR) spectra of NC-LAA, NC-Gly, NC/RGO-LAA, NC/RGO-Gly, and RGO in KBr pellets were recorded using a Thermo Nicolet Avatar 360 FTIR spectrometer (Thermo Fisher Scientific, Waltham, MA, USA) at a spectral resolution of 2 cm⁻^1^ and accumulation of 64 scans.

An AXIS Supra electron spectrometer (Kratos Analytical Ltd. Manchester, UK) using Al Kα radiation, with a photon energy of 1486.6 eV, was used for the XPS measurements. Before the measurements, the samples were stored in an ultra-high vacuum chamber overnight. The binding energies (BEs) were determined with an accuracy of ±0.1 eV using the commercial data processing software ESCApe™ 1.2.0.1325 (Kratos Analytical Manchester, UK). The samples for the analysis were placed on conductive tape. The concentrations of the different chemical elements (in at.%) were calculated by normalizing the areas of the photoelectron peaks to their relative sensitivity factors. The deconvolution of the peaks was performed using the commercial data processing software ESCApe^TM^ from Kratos Analytical Ltd.

### 3.6. CO_2_ Adsorption Experiments

CO_2_ adsorption isotherms were measured using a Quantachrome Nova 1200e (Anton Paar, Boynton Beach, FL, USA) analyzer. Before analysis, the samples were degassed at 200 °C for 18 h. The experiments were conducted with pure CO_2_ gas (99.995%, Messer, Sofia, Bulgaria) at two different temperatures (273 K and 303 K) to calculate the heat of adsorption. The adsorption capacities were determined, and the isosteric heats of adsorption (Qst) were also computed. Predicted selectivity was determined using the Ideal Adsorbed Solution Theory (IAST) from the single-component adsorption isotherms at the same temperatures.

## 4. Conclusions

This paper reports the preparation of two types of nanocarbon and composite graphene-based materials for CO_2_ capture by a one-step, simple, cost-effective, and environmentally benign method, using a green approach with the application of L-ascorbic acid and glycine. XRD analyses revealed the amorphous nature of the prepared NCs and the presence of reduced graphene oxide in the composites. The NCs and NC/RGO composites obtained from two precursors showed quite different morphologies, which is a prerequisite for the observed developed porous structure. A hierarchical porous structure was found for NC-LAA, NC/RGO-LAA, and NC/RGO-Gly. The pore structures of NC/RGO-LAA and NC/RGO-Gly combined the textural features of NC-LAA and NC-Gly with those of RGO, and new textural properties emerged. The NC/RGO-LAA consisted of large graphene sheets, covered with well-defined, small, spherical particles. It was determined that the average size of the particles of both LAA-derived samples was about 11.5 nm, leading to the assumption that these particles were carbon dots or dot-like objects.

At 273 K and 100 kPa, the adsorption capacity of RGO was 1.2 mmol/g. NC-LAA and NC-Gly reached 2.6 mmol/g and 2.5 mmol/g, respectively, while for the composites, the capacities were 1.7 mmol/g for NC/RGO-Gly and 3.5 mmol/g for NC/RGO-LAA. The composites showed enhanced adsorption capacity values, surpassing the simple sum of their components; thus, a synergic effect between the nanocarbons and RGO can be considered. The adsorption ability of the glycine-derived materials is related to the presence of nitrogen-containing functional groups. The heats of adsorption for NC-LAA, NC-Gly, and NC/RGO-Gly reveal chemisorption with CO_2_. Except for chemisorption, the NC/RGO-LAA material showed a sustained physical adsorption up to higher CO_2_ coverage. The best adsorption of CO_2_, observed for NC/RGO-LAA, was due to the combination of the specific morphological forms of carbon dots and RGO that prevents restacking of the graphene sheets, leads to enhanced specific surface area of the composite, and provides large exposure of functional groups of both types of carbon materials.

In conclusion, the composition of two carbon materials in NC/RGO-LAA ensures both sufficient oxygen surface functionalization and a proper hierarchical porous structure, thus providing excellent adsorption properties toward carbon dioxide and enhanced selectivity over nitrogen.

## Figures and Tables

**Table 1:** The CO_2_ adsorption capacities of different adsorbents evaluated at 273 K and 1 bar.

Adsorbent	Adsorption Capacity	Adsorption Mechanism	Reference
Potassium nickel hexacyanoferrate Prussian Blue analogs (K-NiFe-PBAs)	3.0 mmol·g^−^¹	Physical	[12]
N and B-doped graphene aerogels	2.9 mmol·g^−^¹	Not pointed	[15]
Chitosan aerogels with graphene oxide nanosheets	4.14 mmol·g^−^¹	Not pointed	[15]
Biordered ultramicroporous graphitic carbon	7.81 mmol·g^−^¹	Physical	[16]
Reduced graphene	2.36 mmol·g^−^¹	Physical	[16]
Activated carbon	4.66 mmol·g^−^¹	Physical	[17]
CaBTC-derived MOF (CaO/CN-5)	2.30 mmol·g^−^¹	Chemical	[18]
Periodic mesoporous organosilica (PMO) nanoparticles	2.26 mmol·g^−^¹	Physical	[19]
Metal–organic framework	3.7 mmol·g^−^¹	Physical	[20]
MIP-206-OH-Gly MOF	2.15 mmol·g^−^¹	Physical	[21]
Covalent Organic Frameworks (COFs)	3.2 mmol·g^−^¹	Not pointed	[22]
Click-based porous cationic polymer	2 mmol·g^−^¹	Mixed	[23]
Amine-functionalized mesoporous silica	0.7 mmol·g^−^¹	Chemical	[24]
Bifunctionalized mesoporous silica materials	1.22 mmol·g^−^¹	Physical	[25]
Biochar derived from vine shoots	4.07 mmol·g⁻¹	Physical	[26]
Zeolite Na-ZK-4 (2.3)	4.86 mmol·g⁻¹	Physical	[27]
Amine-modified zeolite NaA	80 cm^3^·g⁻¹	Chemical	[28]
Ni(II)/SSZ-13	4.49 mmol·g^−^¹	Mixed	[29]
N-doped activated biocarbon	3.6 mmol·g^−^¹	Physical	[30]
Multi-walled CNTs	0.64 mmol·g^−^¹	Not pointed	[31]
Graphene oxide with 2,6-diformyl-4-methyl phenol	8.10 mmol·g^−^¹	Physical	[32]
Composite of UiO-66-(OH)_2_ and MWCNTs	5.75 mmol·g^−^¹	Physical	[33]
AC CARB 6X12 55	4.53 mmol·g^−^¹	Physical	[34]
Activated carbon Norit RB 4	3.02 mmol·g^−^¹	Physical	[34]
Lotus seed pot-derived N-doped porous carbon	6.2 mmol·g^−^¹	Mixed	[35]

**Table 2:** Texture characteristics of the investigated materials and referent RGO for comparison.

Sample	S, m^2^/g	V, cm^3^/g	D_av_, nm	S_mi_, m^2^/g	S_ext_, m^2^/g	V_mi_, cm^3^/g
NC-LAA	493	0.27	2.2	367	126	0.15
NC/RGO-LAA	487	0.24	2.0	430	56	0.17
NC-Gly	15	0.02	14	-	-	-
NC/RGO-Gly	88	0.12	5.3	63	25	0.03
RGO	45	0.15	9	4	41	0.002

S, specific surface area; V, total pore volume; D_av_, average pore diameter; S_mi_, microporous specific surface area; S_ext_, external specific surface area; V_mi_, micropore volume.

**Table 3:** Chemical composition of the surface.

Sample	C, at%	O, at%	N, at%
NC-LAA	90.2	9.8	-
NC-Gly	64.8	14.8	22.4
NC/RGO-LAA	87.3	12.7	-
NC/RGO-Gly	81.7	16.2	2.1
RGO	93.4	6.6	

## Data Availability

The original contributions presented in this study are included in the article/Supplementary Materials. Further inquiries can be directed to the corresponding author.

## Supplementary Materials

The following supporting information can be downloaded at https://www.mdpi.com/article/10.3390/molecules30112408/s1: Figure S1: XRD patterns of the initial graphite, L-AA, and Glycine.; Figure S2: FTIR spectrum of L-AA.; Figure S3: FTIR spectrum of Glycine; Figure S4: Scheme of preparation of the composite materials.
