# Supplementary material for: Tailored Carbon Nanocomposites for Efficient CO2 Capture
Source: Molecules. 2025 May 30;30(11):2408. doi: 10.3390/molecules30112408 (PMC12156297; doi:10.3390/molecules30112408)
Supplement: Supplementary file 1 [file molecules-30-02408-s001.zip › molecules-3642026-supplementary.pdf]

Supporting Information

Tailored Carbon Nanocomposites for Efficient CO_2 Capture

Diana Kichukova, Tsvetomila Lazarova, Genoveva Atanasova, Daniela Kovacheva* and Ivanka Spassova

Institute of General and Inorganic Chemistry, Bulgarian Academy of Sciences, 1113 Sofia, Bulgaria; kichukova@svr.igic.bas.bg; lazarova@svr.igic.bas.bg; genoveva@svr.igic.bas.bg; didka@svr.igic.bas.bg ; ispasova@svr.igic.bas.bg

* Correspondence: didka@svr.igic.bas.bg;

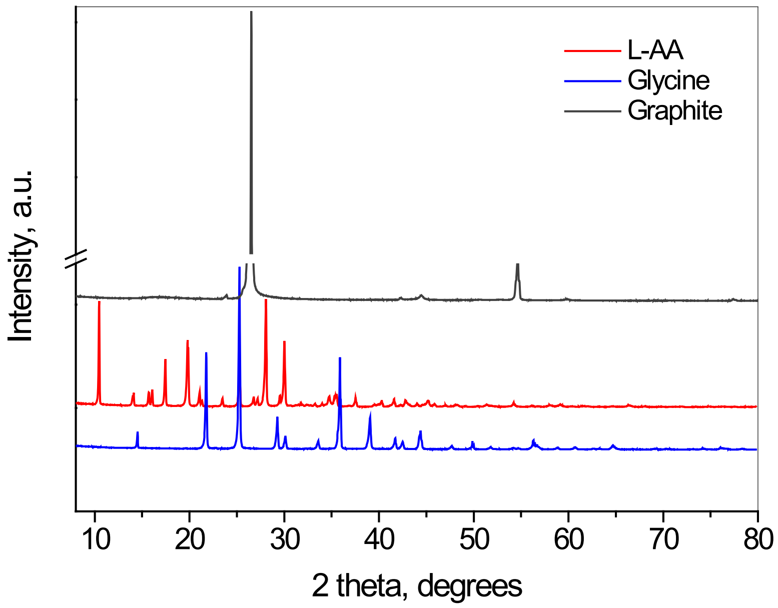

Figure S1. XRD patterns of the initial graphite, L-AA, and Glycine.

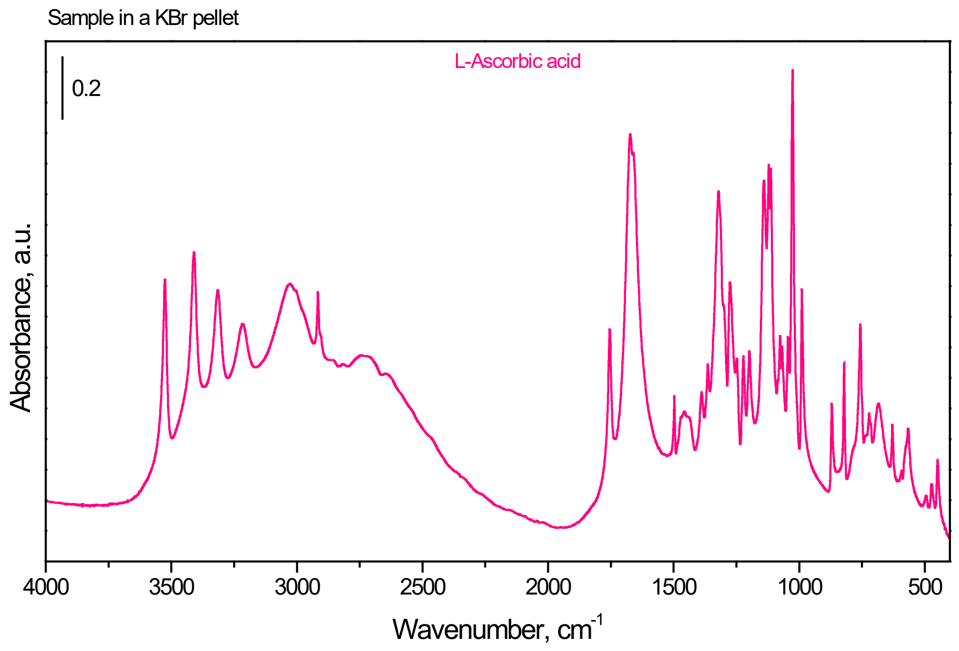

Figure S2. FTIR spectrum of L-AA.

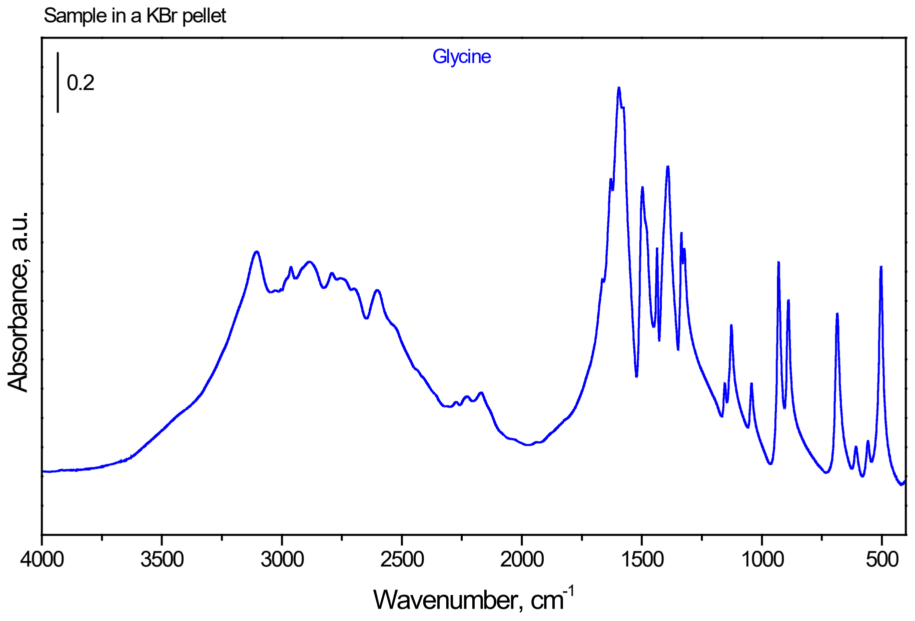

Figure S3. FTIR spectrum of Glycine

The FTIR spectra of the initial L-AA and Glycine are presented in Figure S1 and Figure S2. As for typical highly crystalline organic compounds, the IR spectra consist of many sharp lines. In general, they correspond to the spectra given in the literature [1, 2]. After transformation to NC and NC/RGO materials, some lines characteristic of O-H, C-O, C=O, C-H, N-H, etc., common for all carbon-based materials, are presented in the FTIR of the studied NC and NC/RGO materials.

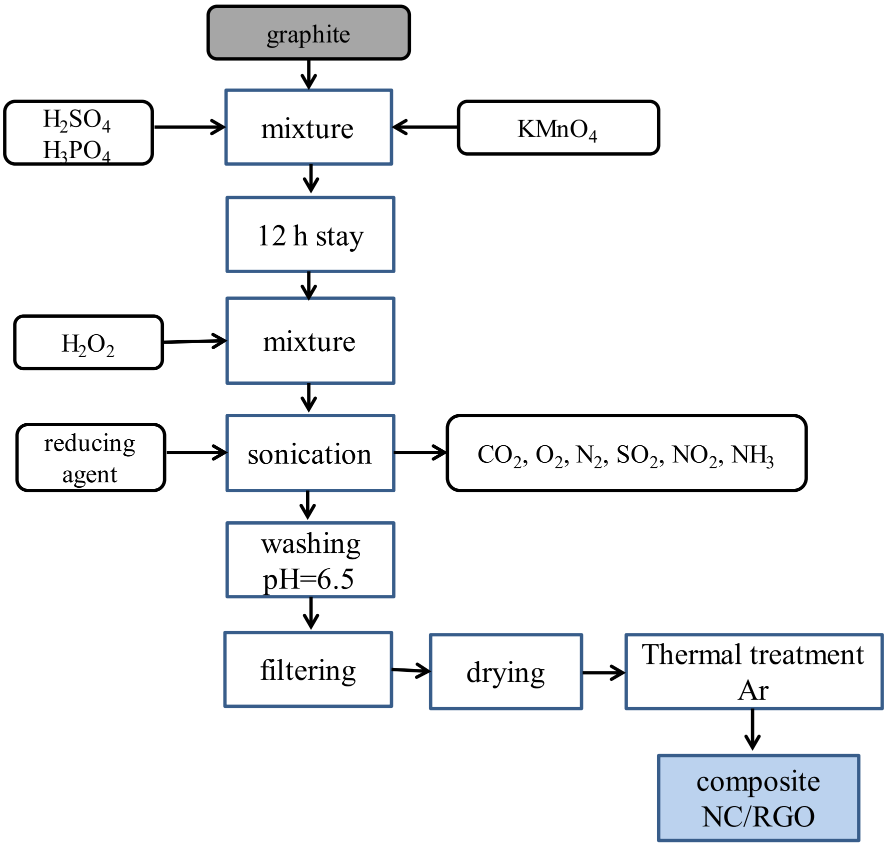

Figure S4. Scheme of preparation of the composite materials

References

1. Farias, M.D.P.; Albuquerque, P.B.S.; Soares, P.A.G.; de Sá, D.M.A.T.; Vicente, A.A.; Carneiro-da-Cunha, M.G. Xyloglucan from *Hymenaea courbaril* var. *courbaril* seeds as encapsulating agent of L-ascorbic acid. *Int. J. Biol. Macromol.* **2018**, *107*, 1559–1566. https://doi.org/10.1016/j.ijbiomac.2017.10.016.
2. Kumar, R.A.; Vizhi, R.E.; Sivakumar, N.; Vijayan, N.; Rajan Babu, D. Crystal growth, optical and thermal studies of nonlinear optical γ-glycine single crystal grown from lithium nitrate. *Optik* **2012**, *123*, 409–413. https://doi.org/10.1016/j.ijleo.2011.04.019.
